# Evolution of *kaiA*, a key circadian gene of cyanobacteria

**DOI:** 10.1038/s41598-021-89345-7

**Published:** 2021-05-11

**Authors:** Volodymyr Dvornyk, Qiming Mei

**Affiliations:** 1 Department of Life Sciences, College of Science and General Studies, Alfaisal University, Riyadh, 11533 Kingdom of Saudi Arabia; 2 Southern Marine Science and Engineering Guangdong Laboratory (Guangzhou), Guangzhou, People’s Republic of China; 3 Key Laboratory of Vegetation Restoration and Management of Degraded Ecosystems, South China Botanical Garden, Chinese Academy of Sciences, Guangzhou, People’s Republic of China

**Keywords:** Molecular evolution, Phylogenetics

## Abstract

The circadian system of cyanobacteria is built upon a central oscillator consisting of three genes, *kaiA*, *kaiB*, and *kaiC*. The KaiA protein plays a key role in phosphorylation/dephosphorylation cycles of KaiC, which occur over the 24-h period. We conducted a comprehensive evolutionary analysis of the *kaiA* genes across cyanobacteria. The results show that, in contrast to the previous reports, *kaiA* has an ancient origin and is as old as cyanobacteria. The *kaiA* homologs are present in nearly all analyzed cyanobacteria, except *Gloeobacter*, and have varying domain architecture. Some Prochlorococcales, which were previously reported to lack the *kaiA* gene, possess a drastically truncated homolog. The existence of the diverse *kaiA* homologs suggests significant variation of the circadian mechanism, which was described for the model cyanobacterium, *Synechococcus elongatus* PCC7942. The major structural modifications in the *kaiA* genes (duplications, acquisition and loss of domains) have apparently been induced by global environmental changes in the different geological periods.

## Introduction

Circadian rhythms or internal biological clock appeared in cells of living organisms as the main tool for adaptation to day–night change caused by the rotation of our planet around its axis^1^. This mechanism controls timely gene expression of a significant part of a genome.

Adaptation to the daily light cycles makes an important contribution to the ecological plasticity of cyanobacteria and apparently confers a selective advantage^2, 3^. It seems particularly important for marine cyanobacteria, which are characterized by ecological niche partitioning^4^.

Cyanobacteria were the first prokaryotes shown to have the circadian system^5^. The circadian system of cyanobacteria has been comprehensively studied in a model strain *Synechococcus elongatus* PCC7942. Its key structural and functional element, central oscillator, consists of three genes: *kaiA*, *kaiB*, and *kaiC*^6^. The corresponding proteins interact with each other: KaiB weakens the phosphorylation of KaiC^7^, while KaiA inhibits dephosphorylation of KaiC by binding to its respective domains^8^.

While the role of KaiA in the cyanobacterial circadian mechanism has been extensively studied (see^9^ for review), the knowledge about its evolution is limited. The first and most comprehensive study so far was published in 2003^10^ and was based on then available GenBank collection of genomic sequences. It suggested the origin of the *kaiA* gene about 1000 Mya. The growing volume of available genomic data allowed for updating the initially proposed evolutionary scenario for the cyanobacterial circadian system and move the *kaiA* origin back to 2600–2900 Mya^11^.

The rapid growth of genomic databases during the last decade prompted for a new, more comprehensive analysis and, respectively, update of the existing evolutionary scenario for *kaiA* and the other circadian genes. The present study analyzed the occurrence, domain architecture, genetic variation and phylogeny of the *kaiA* gene homologs. We attempted to reconstruct the evolutionary history and to determine the evolutionary factors that have been operating on this key genetic element of the cyanobacterial circadian oscillator and might contribute to its function. We also updated a timeline for key events in the evolution of both *kaiA* and the whole circadian system. This study provides new data about the probable functional significance of various residues and motifs in the KaiA protein, and significantly updates our knowledge about the evolution of the cyanobacterial circadian system as a whole.

## Results

### Occurrence and domain architecture of the kaiA genes and proteins in cyanobacteria

**Figure 1:**
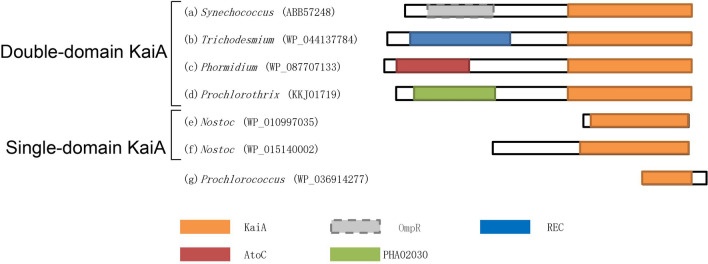
The domain architecture of KaiA proteins. (**a**) *Synechococcus* (ABB57248); (**b**) *Trichodesmium* (WP_044137784); (**c**) *Phormidium* (WP_087707133); (**d**) *Prochlorothrix* (KKJ01719); (**e**) *Nostoc* (WP_015140002); (**f**) *Nostoc* (WP_010997035); (**g**) *Prochlorococcus* (WP_036914277). Homology to the OmpR domain is weak and denoted by dashed box.

Homologs of *kaiA* occur in nearly all major cyanobacterial taxa available in GenBank, including Oscillatoriophycideae, Synechococcales, Pleurocapsales, Spirulinales, Chroococcidiopsidales, and Nostocales. All KaiA proteins can be roughly classified by their architecture into two main subfamilies, single-domain and double-domain, respectively (Fig. 1). The double-domain KaiA (ddKaiA) is about 300 aa long and found in Oscillatoriophycideae, Synechococcales, Pleurocapsales and Spirulinales. The KaiA proteins in *Chroococcidiopsidales* and *Nostocales* feature a single domain (sdKaiA) and are quite variable in length mostly ranging from 89 to 202 amino acid residues (Table S1). However, in some *Nostocales*, such as in *Richelia intracellularis* HH01, sdKaiA experienced even more drastic truncation, up to 45 amino acid residues. Both single-domain and double-domain versions share the conserved KaiA domain (pfam07688), which is the only member of superfamily cl17128.

The BLAST search of the GenBank database also returned several short proteins manifesting high homology to other segments of the KaiA domain. For example, the proteins from two cyanobacterial strains annotated as *Cyanobacteria bacterium QH_1_48_107* and *Cyanobacteria bacterium QS_7_48_42*, possess the KaiA homologs of 56 residues long (PSO52447.1 and PSP04869.1, respectively), which match a region between positions 169 and 224 in the *bona fide S. elongatus* PCC7942 protein (hereinafter the position numbers refer to the *bona fide* KaiA sequence). Interestingly, both these strains possess KaiC but lack KaiB.

Another example is the KaiA homologs found in some *Prochlorococci* (Table S1). They vary from 62 to 66 residues in length and, unlike the previous ones, match residues 238–284 in the respective *S. elongatus* PCC7942 protein (Fig. 1e). In contrast to the above-mentioned two strains, *Prochlorococci* do possess both KaiB and KaiC.

Several strains of *Prochlorococcus* sp. (e.g., MIT9303, MIT9313, and MIT1306) possessed a gene located in the genomic region usually occupied by the *kaiA* gene in the syntenic *bona fide kaiABC* operon, i.e. between the *rplU* and *kaiB* genes. However, unlike *kaiA*, this gene is located on the reverse complement strand. This gene was previously described as a pseudogene in MIT9303 and MIT9313^12^. However, this is apparently not so: according to the genomic annotations, the gene is apparently translated, because it contains an open reading frame and thus may be functional. The putative respective proteins were about the same length (65–132 aa) as the sdKaiA homologs in other cyanobacteria. However, these proteins showed no apparent homology to either KaiA or any other proteins in the non-redundant NCBI protein database according to the BLAST search. Their function remains unknown.

In addition to cyanobacteria, the KaiA protein was found in other marine and freshwater bacteria, e.g., *Propionibacteriaceae bacterium* and *Planctomycetaceae bacterium* TMED241 (Table S1). This finding is unlikely an artefact, because screening of this species’ genome assembly revealed the full syntenic *kaiABC* operon typically found in cyanobacteria.

According to the Conserved Domain Database^13^, the N-terminal domain of the *bona fide* ddKaiA protein of *S. elongatus* PCC7942 belongs to the OmpR family. However, the observed homology is quite weak and was detected only when a lower E-value was applied. OmpR is a DNA-binding dual transcriptional regulator and is often an element of various two-component regulatory systems. While most ddKaiA proteins share the above architecture, few of them manifest some variability by featuring other domains instead of OmpR, namely REC, AtoC or PHA02030 (Fig. 1). However, regardless of the domain architecture, all KaiA homologs appear to form a homodimer in solution^14, 15^. On the other hand, this may not be the case for the truncated homologs.

The *kaiA* genes in some species are annotated as pseudogenes as, for example, in *Aphanizomenon ovalisporum* (CDHJ01000032, locus tag apha_00336). The functional deficiency of their KaiA might result by lack of the N-terminal fragment.

### Conserved residues of possible functional significance

**Figure 2:**
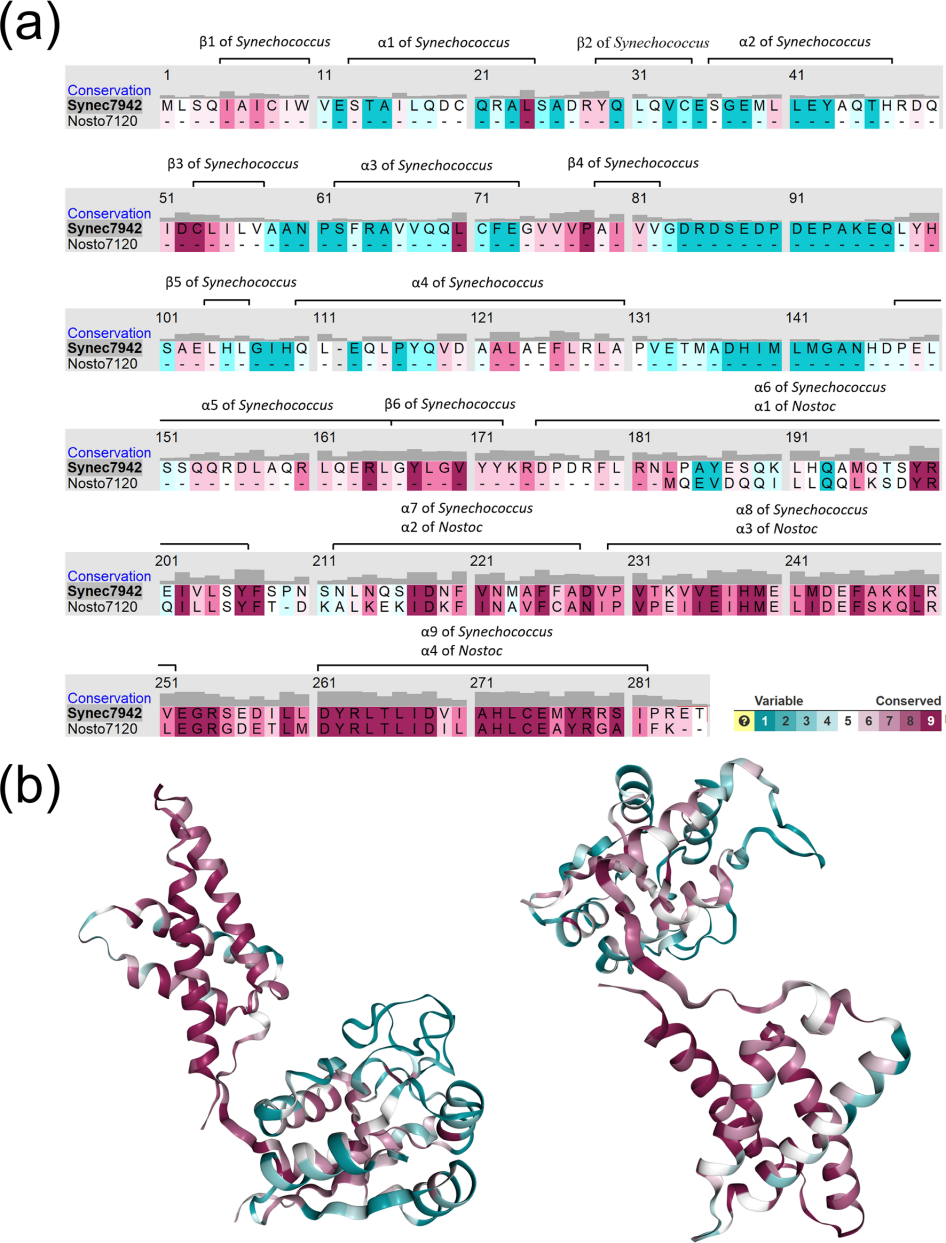
Group-conserved residues identified by ConSurf. Degrees of conservation in subfamilies were visualized by Chimera v.1.10.2^17^. (**a**) Conserved sites of the KaiA protein. Number the residues is accordant with the *Synechococcus* KaiA (ABB57248). The black bars above sequence indicate the level of conservation (1–9). (**b**) Conserved sites labeled (red) in the 3D structure of the *Synechococcus elongatus* KaiA protein (PDB: 1R8J_A) (left: N-terminal region; right: KaiA domain).

The C-terminal domains of the single-domain and double-domain KaiA homologs have quite similar structure consisting of four conserved helices. The most terminally located helix (α4 of *Nostoc* and α9 of *Synechococcus*, sites 259–280) has the highest level of conservation (Fig. 2) and acts as a dimer interface^14^. However, the KaiA proteins in some species (e.g., *Trichormus variabilis* ATCC 29413) have a significantly shorter C-terminal region and lack at least two most terminally located KaiC-binding residues. In *Synechococcus elongatus* PCC 7942, the truncation of C-terminal amino acid residues leads to the shortened circadian periods because the binding between KaiA and KaiC is strengthened^16^.

The comparative analysis of the KaiA homologs identified 27 sites in the namesake domain universally conserved in most cyanobacteria according to the BLOSUM62 matrix^18^. Five sites, M241, D242, E251, L265, and D267, are 100% conserved across all cyanobacteria, including the truncated KaiA homologs in the *Prochlorococcus* sp. and *Trichormus variabilis* ATCC 29413 (Table 1). Ten of the 27 conserved sites are fixed, which strongly suggests their high functional significance. However, the respective data are available only about five of them. The known functions of the conserved sites are related to either the maintenance of the KaiA homodimer structure^14^ or binding the KaiC protein^19^.

**Table 1:** A list of the universally conserved positions in the KaiA homologs of cyanobacteria with the reference to the *bona fide* protein of *S. elongatus* PCC7942.

Position number	Amino acid in *S. elongatus* PCC7942	Possible variants in other cyanobacteria	Effect of mutation or putative function	References
198	Y	None	Unknown	na
201	I	L, V	Unknown	na
202	V	L, I	Unknown	na
205	Y	None	Unknown	na
206	F	Y	Unknown	na
216	I	M, L, V	Unknown	na
217	D	E	Unknown	na
224	F	Y	Abolishes the rhythm	^18^
234	V	I, L, M	Unknown	na
237	H	None	Unknown	na
241^a^	M	I, V	Modifies amplitude KaiC binding site	^19^ ^14^
242^a^	D	E	Modifies amplitude KaiC binding site	^19^ ^14^
251^a^	E	K	Unknown	na
258	L	I, V	Unknown	na
260	D	None	Dimer interface site	^14^
261	Y	None	Unknown	na
262	R	None	Dimer interface site	^14^
265^a^	L	I, V	Unknown	na
266	I	M, L, V	Modifies amplitude, KaiC binding	^19^
267^a^	D	None	Unknown	na
269	I	M, L, V	Dimer interface site	^14^
270	A	S	Dimer interface site	^14^
271	H	N	Unknown	na
272	L	M	Dimer interface site	^14^
274	E	None	Dimer interface site	^14^
276	Y	None	Dimer interface site	^14^
277	R	None	Dimer interface site	^14^

^a^Conserved in the truncated KaiA too.

### Functional divergence of the KaiA homologs

The analysis of the functional divergence between the single-domain and double-domain KaiA proteins showed the significantly altered functional constraints (rates of evolution) after duplication of the ancestral gene. On the other hand, no type II functional divergence (radical amino acid changes without a rate shift) was detected. In the analyzed segment of 96 amino acid residues (nearly the full length KaiA domain), the effective number of the type I residues was 33. That means, nearly 1/3 of the domain experienced significant shift in evolutionary rate.

### Nucleotide diversity and selection of kaiA

The C-terminal region of dd*kaiA* is more variable (*d*_N_ = 0.30 ± 0.03, π = 0.36 ± 0.00) as compared to the single-domain homologs (*d*_N_ = 0.20 ± 0.02, π = 0.26 ± 0.01) and is much more conserved than the N-terminal one (*d*_N_ = 0.88 ± 0.06, π = 0.52 ± 0.00). This may be due to the evolutionary younger age of sdKaiAs as compared to ddKaiAs (*Nostocales* are evolutionary younger than *Oscillatoriophycideae*, *Synechococcales* and *Pleurocapsales*)^10^ or/and because of the higher functional significance of the C-terminal region of KaiA (binds to KaiB and KaiC)^20^. Besides, the N-terminal domains of the dd*kaiA* genes may vary and manifest functional diversity (Fig. 1). None of the applied methods detected positive selection in the *kaiA* genes.

### The phylogeny of the kaiA genes and time estimates of the evolutionary events

**Figure 3:**
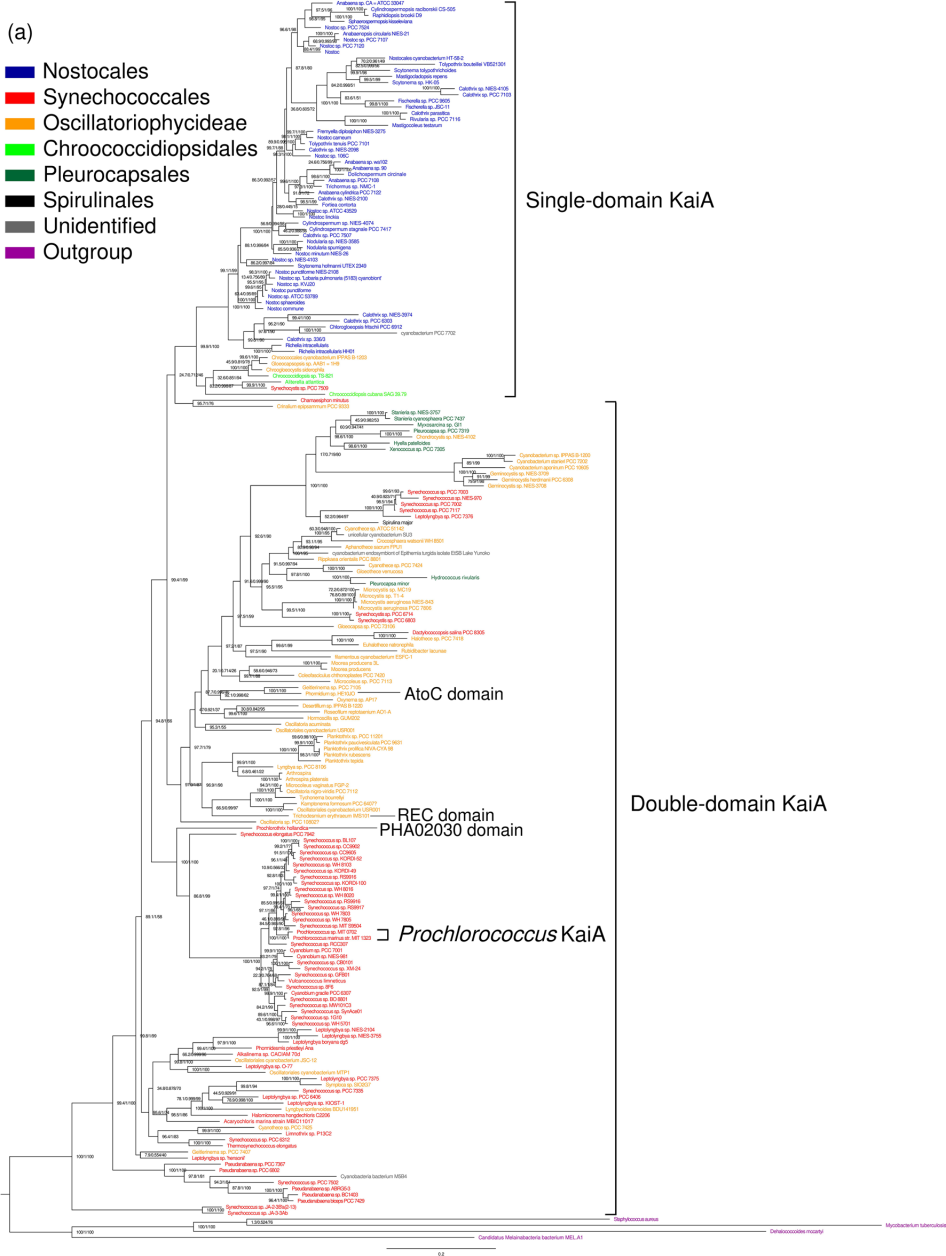

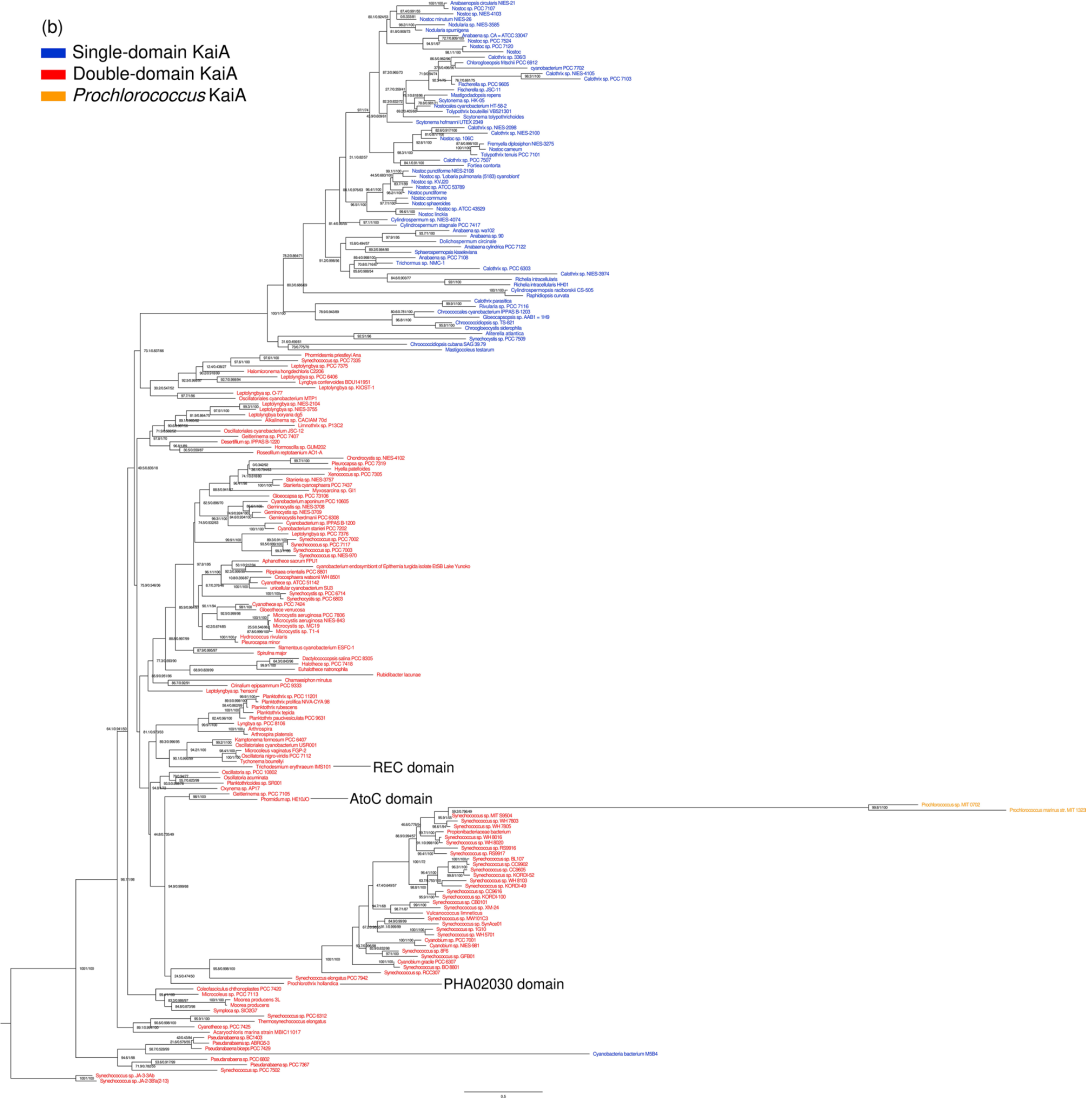
The maximum-likelihood phylogenetic trees of: (**a**) the 16S and 23S rRNA genes (species tree) and (**b**) KaiA homologs (gene tree). The node support values are ultrafast bootstrap/SH-aLRT branch test/approximate Bayes test.

The comparison of the species and gene trees clearly shows they are largely incongruent. Both trees feature several monophyletic clades with a strong statistical support (Figs. 3 and S1). These few clades in both trees match each other by set of taxa, but their positions in the overall tree topologies are quite different, and so are the positions of taxa within the clades. The ML trees manifested better resolution than the Bayesian trees, particularly at deeper nodes. The comparative analysis of the species and gene trees identified 36 lateral gene transfers having occurred in the evolution of the *kaiA* genes (Supplementary file 1, Fig. S2). However, while the lateral transfers seem to be quite common within the clades, they have been much less frequent or absent between the clades. Furthermore, no HGTs were detected between the taxa with single-domain and double-domain *kaiA* genes. The results of the HGT analysis suggest that heterocystous cyanobacteria, which are a group possessing the sd*kaiA*, have experienced the most frequent transfers (Fig. S2).

The time estimates of the major events in the evolution of the *kaiA* homologs are provided in Table 3. Both Bayesian and ML estimates are similar and suggest three main periods when these events probably occurred: about 30–100, 500–600, and 1000–1500 Mya. The origin of the sd*kaiA* was apparently associated with the origin of *Chroococcidiopsidales* that occurred about 1500 Mya.

### The 3D structure of the KaiA homologs

**Figure 4:**
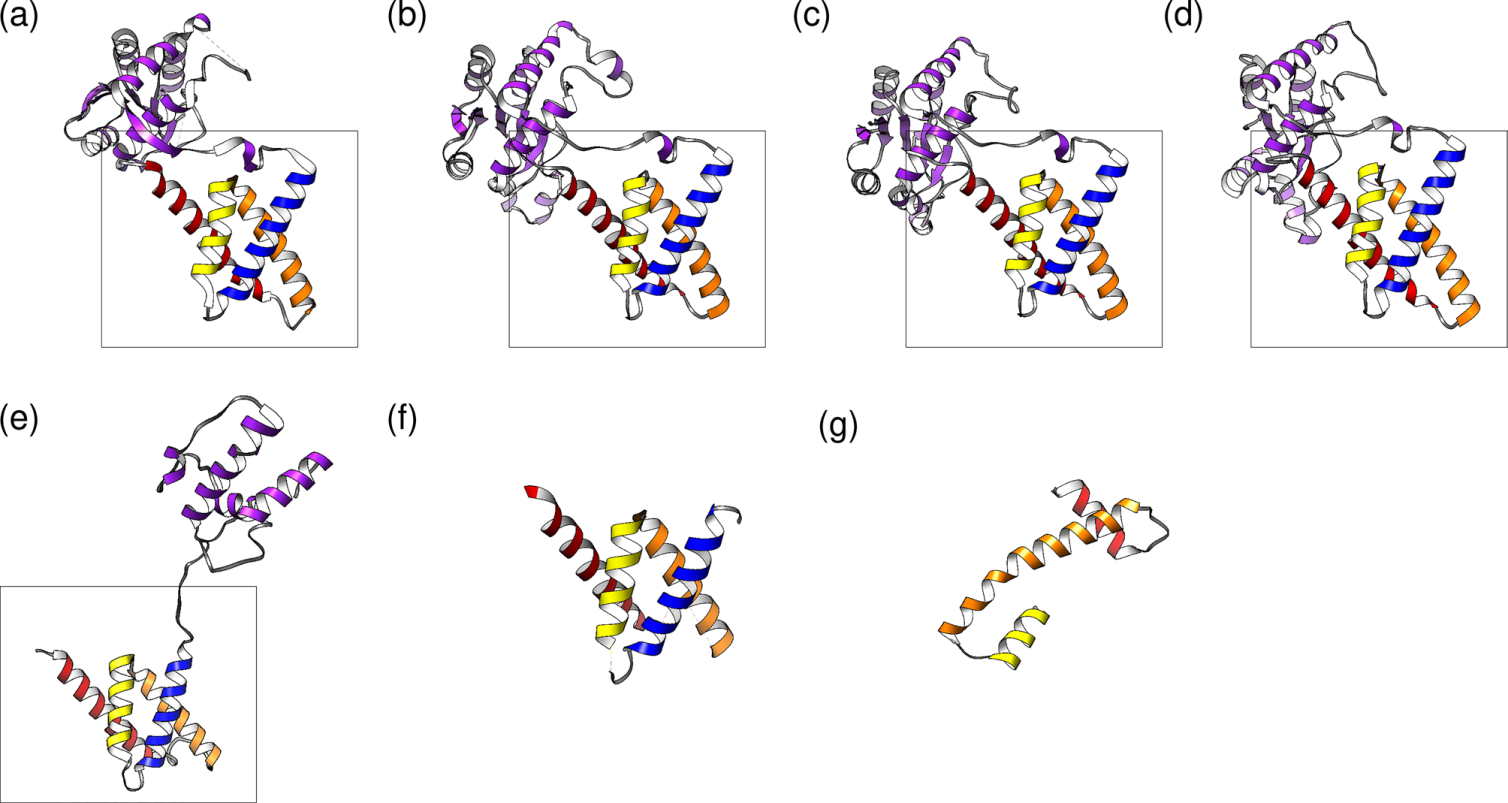
Models of the 3D structure of the KaiA homologs from different cyanobacteria. (**a**) *Synechococcus* (ABB57248, PDB: 1R8J); (**b**) *Trichodesmium* (WP_044137784); (**c**) *Phormidium* (WP_087707133); (**d**) *Prochlorothrix* (KKJ01719); (**e**) *Nostoc* (WP_015140002); (**f**) *Nostoc* (WP_010997035, PDB: 1R5Q); (**g**) *Prochlorococcus* (WP_036914277). The KaiA domain is boxed. Models (**a**) and (**f**) are experimental, the others are computer generated.

The in silico inferred 3D models of the KaiA homologs have essentially the same structure as those determined experimentally (Fig. 4). They all feature a highly conserved KaiA domain formed by four pairwise paralleled helices. The only exception is a KaiA homolog of *Prochlorococcus* sp. (Fig. 4g). It is truncated and features three helices, two of which on the termini are short. The long helix, however, is highly homologous to the terminal helix (α9) of the KaiA domain in the *bona fide* protein of *S. elongatus* PCC7942 (Fig. 2a).

## Discussion

### The occurrence and distribution of kaiA among cyanobacterial taxa suggest an ancient origin of the gene

The *kaiA* genes were found in all analyzed cyanobacteria except *Gloeobacter*. The latter is thought to be the most ancient cyanobacterium, which, while being able for photosynthesis, lacks a few structures and genes common for all other cyanobacteria^21^. Our results on the *kaiA* occurrence are essentially in agreement with those recently reported by Schmelling et al.^22^ who performed comprehensive screening of prokaryotes for circadian orthologs. In addition, the present study firstly reports the *kaiA* homologs and the whole *kaiABC* operon in prokaryotes other than cyanobacteria. The most probable explanation of this may be a lateral transfer of the operon from cyanobacteria.

The truncated *kaiA* homologs from *Prochlorococcales* were not reported by the early evolutionary studies of the circadian system in cyanobacteria (see, e.g.^10, 11^). This might be due to the much smaller volume of then available genomic data and poor annotations of genomes.

The occurrence of the *kaiA* homologs across all cyanobacterial taxa suggests that this gene is of ancient origin, probably as old as most cyanobacteria themselves. Indeed, *kaiA* was found in the thermophilic strains from Yellowstone, *Synechococcus* sp. JA-2-3B'a(2-13) and *Synechococcus* sp. JA-3-3Ab, which are located at the root of the cyanobacterial phylogenetic subtree (Figs. 3a and S1a). It might be that the gene was horizontally transferred from the evolutionary younger lineages. However, no such transfers to this clade was detected (Fig. S2).

In the pioneering study about origin and evolution of the cyanobacterial circadian genes, it was hypothesized that *kaiA* originated about 1000 Mya after two other key circadian genes, *kaiB* and *kaiC*^10^. This hypothesis was later revisited based on the growing available genomic data and much earlier origin of the *kaiA* gene was suggested^23, 24^. This revision is further supported by the results of the present study.

### The domain architecture underlies evolutionary and functional constraints of the kaiA genes

All *kaiA* genes can be divided into two large groups according to their domain architecture: single-domain and double-domain, respectively. The occurrence of these two versions of the gene is taxon-specific (Figs. 3 and S2). The sd*kaiA* occurs exclusively in *Chroococcidiopsidales* and *Nostocales*, while dd*kaiA* was found across all other cyanobacterial taxa. However, the N-terminal domain in dd*kaiA* varies quite significantly (especially as compared to the *kaiA* domain) across cyanobacteria and its homology to OmpR is quite weak. This suggests that the ancestral OmpR domain has been under weak selective constraints in the course of evolution that might result in its functional modification or even loss of the original function.

The truncation of the ancient dd*kaiA* into sd*kaiA* and the origin of *Chroococcidiopsidales* were apparently associated with each other (Table 2, Fig. 3). The loss of the N-terminal domain probably conferred evolutionary constraints to the *kaiA* domain: it is significantly less variable in sd*kaiA* than in dd*kaiA* (Table 2). Another evidence comes from the analysis of HGTs: while the transfers have been quite common within the clades of the genes with the same architecture (i.e., either single-domain or double-domain, respectively), no HGTs were determined between these clades (Fig. S2).

**Table 2:** Patterns of nucleotide diversity in the *kaiA* homologs of cyanobacteria.

	*d*_N_			π		
	N-terminal region	*kaiA* domain	Average over gene	N-terminal region	*kaiA* domain	Average over gene
dd*kaiA*,	0.78 ± 0.05	0.35 ± 0.04	0.57 ± 0.03	0.48 ± 0.00	0.38 ± 0.00	0.42 ± 0.00
sd*kaiA*	0.79 ± 0.05	0.26 ± 0.02	0.31 ± 0.02	0.52 ± 0.02	0.27 ± 0.02	0.27 ± 0.01
Average over domain	1.08 ± 0.06	0.37 ± 0.03	0.54 ± 0.03	0.57 ± 0.01	0.33 ± 0.01	0.33 ± 0.01

The rate of synonymous nucleotide substitutions (*d*_S_) was not estimated due to saturation.

The KaiA domain of the protein is a key player in its binding to KaiC: several functionally important or critical residues have been identified experimentally in this domain^14, 19^. However, there are several more highly conserved or invariable residues identified in the present study (Table 1), which are apparently functionally important, but their exact function has yet to be determined.

There are several factors, which likely confer evolutionary constraints to the *kaiA* genes and limit HGTs even between the clades with the same domain architecture. In particular, this may be related to possible interaction with other elements of the circadian system. For example, some studies showed that KaiA competes with CikA in binding to KaiB and phosphorylation of KaiC^25^. However, this mechanism is likely not universal, because *bona fide* CikA is absent in many cyanobacteria^11, 22^. Therefore, the observed variation in the *kaiA* domain and, respectively, above mentioned constraints may be related to functional modifications of KaiA to adjust to the circadian input pathway alterations. Wood et al.^26^ reported that KaiA of *S. elongatus* PCC7942 binds the quinone by its N-terminal domain (OmpR, Fig. 1). This interaction helps to stabilize KaiA and is important for the mechanism of the KaiC phosphorylation. However, the sdKaiA proteins either lack the N-terminal domain completely or have it truncated (Fig. 1e–g) that means the circadian system in *Chroococcidiopsidales* and *Nostocales* should either lack this binding ability completely or have a different one. Furthermore, some cyanobacterial lineages have different N-terminal domains (Fig. 1) that assumes the different (if any) interaction with the quinone.

### The variation patterns in the kaiA gene and the encoded protein support the functional diversification of the circadian system in cyanobacteria

There is ample evidence that the cyanobacterial circadian system has experienced extensive evolutionary diversification (see, e.g.^11, 24^ for review). The results of the present study provide further support for that. Not only did the functional divergence occur between the single-domain and double-domain KaiA proteins, but also it occurred between the clades within these two subfamilies (data not shown).

In its native state, KaiA is a dimer whose only known function is binding to KaiC CII domain and inducing its autophosphorylation^27, 28^. Therefore, in the circadian system missing KaiA, the timing mechanism may be simplified as it was suggested for *Prochlorococcus*^29^. However, it seems that even within the *Prochlorococcus* lineage, different versions of the simpler circadian system may exist. Indeed, as the results of the present study suggest, some *Prochlorococcus* strains possess, albeit truncated, but a highly conserved homolog of *kaiA* (Fig. 1). This extreme conservation, particularly at the functionally important residues common for the KaiA homologs across cyanobacteria, may suggest that the function of this truncated KaiA is somewhat similar to that of the *bona fide* protein. Strains of *Prochlorococcus* are known for their niche-specific adaptation, particularly with respect to the different light and temperature regimes, and extensive diversification into many co-existing ecotypes^30^. The presence/absence of the *kaiA* homolog or its orphan replacement may be associated with this adaptation. For example, strains MIT9303 and MIT9313, which possess the orphan gene, were reported as adapted to low light^30^. Importantly, despite the quite significant type I functional divergence (altered evolutionary rate), no type II divergence (radical amino acid changes) was detected in the KaiA domain of the truncated homologs. In these terms, it would be interesting to determine the exact functional significance of the universally conserved residues identified in the present study (Table 1).

### Phylogenetic dating supports the hypothesis about the association of the circadian system evolution with the geochronological events

The origin of the *kaiA* gene was initially estimated about 1000 Mya based on then available genomic data^10^. Since then, as more data has been accumulated, this estimate has been reconsidered^23, 24^. The results of the present study suggest that the *kaiA* gene is evolutionarily much older than it was thought before and its origin can be dated back to that of most cyanobacteria, i.e., about 3000 ± 500 Mya depending on the estimation methods.

The loss of *kaiA* in *Prochlorococcales* was apparently associated with the origin of this taxon that occurred about 150–200 Mya (Table 3). This estimate is in broad agreement with the previously reported dating based on the rRNA sequences^31^. Holtzendorff et al.^12^ hypothesized that *kaiA* might experience a stepwise deletion in *Prochlorococcales* by referring to the “*kaiA* pseudogene” in strains MIT9303 and MIT9313 as the evidence. However, the results of the present study suggest an alternative scenario: the original *kaiA* gene was initially either lost in *Prochlorococcales* or replaced by the orphan gene (the one erroneously referred to as the *kaiA* pseudogene). This assumption seems quite feasible given that the above two strains as well as others missing the truncated *kaiA* homologs belong to the earliest branching low-light adapted clades of the *Prochlorococcus* subtree^32, 33^.

**Table 3:** Bayesian and maximum-likelihood time estimates for the events in the evolution of the *kaiA* homologs based on the species trees (Mya).

Evolutionary events	Bayesian^a^	Maximum likelihood^b^
HGT of *kaiA* from *Synechococcus* to *Prochlorococcus* followed by truncation	34.2–100	31.7–145.6
Loss of dd*kaiA* in *Prochlorococcus*	154.1 (91.1, 222.0)	202.5 (161.0, 262.2)
Domain fusion of AtoC in *Phormidium*	508.8 (134.5, 1023.4)	709.3 (522.2, 971.0)
Domain fusion of PHA02030 in *Prochlorothrix hollandica*	1032.6 (673.0, 1422.8)	1363.4 (1151.6, 1605.9)
Domain fusion of REC in *Trichodesmium erythraeum*	1405.9 (1313.9, 1498.5)	1513.6 (1314.6, 1752.5)
Origin of sd*kaiA*/*Chroococcidiopsidales*	1483.3 (1325.5, 1683.4)	1650.8 (1530.2, 1808.7)
CP1: origin of *Nostocales*	1300–1480	
CP2: origin of cyanobacteria	3000	

^a^Posterior mean (95% HPD).

^b^Mean (95% CI).

After that, about 50–100 Mya, the *kaiA* gene was laterally transferred from the *Synechococcus* lineage to some *Prochlorococcales* and underwent a drastic truncation (Fig. 1e, Table 3). One more scenario may be based on the fact that the truncated *kaiA* homologs are apparently common in various *Synechococcales* and *Nostocales* and therefore the truncation might occur prior to the HGT to *Prochlorococcales*. These HGT and follow-up truncation (if any) might be related to the Cretaceous–Paleogene (K–Pg) extinction, which occurred about 66 MYA due to the asteroid impact having caused global ecological devastation, including rapid acidification of the oceans and light regime change^34, 35^.

There were several major structural changes in the *kaiA* genes (Table 3). The origin of sd*kaiA* and *Chroococcidiopsidales* falls within the Calymmian Period, the first geologic period in the Mesoproterozoic Era about 1500 MYA. These events might be associated with oxygenation of the Metaproterozoic ocean that occurred about 1570–1600 Mya^36^. The domain fusion in *kaiA* of *Phormidium* occurred about 500–700 Mya, which corresponds to either the Ediacaran Period known for its Avalon explosion^37^ or the Cambrian explosion^38^.

Of course, the above interpretation of the obtained estimates has some limitations, one of which is the uncertainty of the fossil calibrations. On the other hand, the dates of the multiple events in the evolution of the *kaiA* genes inferred by the molecular methods match well the specific events in the Earth geochronology, which indeed might affect this evolution.

## Conclusion

The present study provides compelling evidence for the ancient origin of the *kaiA* gene and thus revises the previously suggested timeline of the cyanobacterial circadian system evolution. It also prompts for further experimental studies to determine the exact functions of the identified universally conserved/fixed residues in the KaiA domain.

## Materials and methods

### DNA and protein sequences

The sequences of the KaiA proteins and respective genes were retrieved from the GenBank using the KaiA sequence of *Synechococcus elongatus* PCC7942 (WP_011377921) as a query. We utilized the genomic BLASTP^39^ to search the database. Only the sequences from the fully sequenced cyanobacterial genomes were used for the analyses. Bit score of 100 was applied as a cutoff value for sequence selection. Finally, the sequences from 226 strains were retained for the analysis. The used sequences are listed in Supplementary Table S1.

Besides, we used the 16S and 23S rRNA genes for the construction of the species tree. The respective DNA sequences of *Acaryochloris marina* strain MBIC11017 (CP000828) and *Nostoc* sp. PCC 7107 (CP003548) were used as the probes. In addition to the rRNA genes of cyanobacteria, the respective sequences of *Staphylococcus aureus*, *Dehalococcoides mccartyi*, *Mycobacterium tuberculosis*, and *Candidatus Melainabacteria bacterium MEL.A1* were retrieved for the phylogenetic analysis (Table S1). In total 231 sequences were used in the analyses.

### Sequence editing and alignment

The full protein sequences were aligned using the combined sequence and structure-based algorithm implemented in the PRALINE server^40, 41^; the nucleotide sequences were aligned according to the protein alignment by Rev-Trans v.1.4 (http://www.cbs.dtu.dk/services/RevTrans/)^42^. The rRNA sequences were aligned using MAFFT^43^. The aligned sequences were inspected visually and trimmed manually to remove poorly aligned regions and thus to improve a phylogenetic signal. The resulting final alignment of the KaiA protein subfamilies included 296 positions; the concatenated 16S-23S rRNA alignment counted 2941 positions.

### Identification of conserved residues

The ConSurf server (http://consurf.tau.ac.il/) was utilized to identify group-specific conserved sites in the KaiA proteins^44^. The analysis was conducted using a Bayesian procedure, the JTT substitution matrix, and *Synechococcus elongatus* KaiA (SMTL ID: 4G86_A) as a template^45^.

### Analysis of nucleotide diversity and selection

The *d*_N_ values of the *kaiA* genes were calculated using the modified Nei-Gojobori method (with Jukes-Cantor correction and 1000 bootstrap replicates)^46^ as implemented in MEGA X^47^. To test the saturation of synonymous substitutions, pairwise *d*_S_ estimates were calculated first. Most pairwise *d*_S_ values were above 2, thus indicating that synonymous nucleotide substitutions were saturated. Also, the nucleotide diversity of the KaiA was analyzed using DnaSP v. 6.12.03^48^. The level of variation was estimated by π^49^.

Positive selection in the *kaiA* genes was analyzed using several approaches implemented in the Datamonkey server^50^. Site-specific positive selection was analyzed using FUBAR^51^; the branch-site positive selection was tested using aBSREL^52^. A gene-wide test for positive selection was conducted using BUSTED^53^.

### Analysis of the functional divergence

The functional divergence between the KaiA subfamilies at the namesake domain was analyzed using the DIVERGE3 software^54^. The following parameters were estimated: type I and type II functional divergence^55, 56^, effective number of sites related to this divergence. The False Discovery Rate (FDR) of the probability cut-off for the predicted sites was set at < 0.05.

### Phylogenetic analysis

Using only the KaiA domain for the phylogenetic inference yielded a poorly resolved tree. Therefore, the full KaiA protein alignment of 296 positions was utilized. The maximum-likelihood phylogenetic analysis was conducted using the IQ-TREE software^57^ with the built-in ModelFinder function^58^. Based on the ModelFinder analysis results, the JTT model^59^ with a gamma distribution (α = 0.840) was used for the phylogenetic analysis of the KaiA homologs; the GTR model with a proportion of invariable sites and gamma distribution (GTR + I + 4G, p-inv = 0.106, α = 0.662) was applied to the analysis of the rRNA genes. The node support was inferred according to the ultrafast bootstrap^60^, SH-aLRT branch test^61^, and approximate Bayes test^62^.

The Bayesian relaxed clock as implemented in BEAST v.2.6.2^63^ was used to construct phylogenetic trees. The length of the MCMC chain was set for 100 million with trees sampling every 1,000 steps. The maximum clade credibility tree was determined using TreeAnnotator v.1.7.5 from the BEAST software package.

The horizontal gene transfers were determined using the bipartition dissimilarity algorithm implemented in the HGT-Detection software^64^.

### Time estimates for the evolutionary events

Two internal calibration points (CP1 & CP2) based on cyanobacteria fossil evidence were used for evolutionary time estimates. CP1 indicated the origins of Nostocales (1480–1300 Mya)^65^, CP2 corresponded to the lower boundary of the estimates for the origin of cyanobacteria and was limited to the mid-Archean, before the Great Oxidation Event (~ 3000 Mya)^66^. The height of the whole tree was constrained to 4000 Mya. The computations were conducted using BEAST v.2.6.2^67^ and IQ-TREE^57^ as mentioned above.

### Three-dimensional modeling of the KaiA proteins

The predicted 3D models of KaiA proteins with the different domain architecture were constructed and refined using the respective methods implemented in the GalaxyWEB server^68^. The quality of the models was assessed by the structure assessment tool of the SWISS-MODEL server^69^.

## Supplementary Information

Supplementary Information.

Supplementary Table S1.

## Data Availability

All data generated or analyzed during this study are included in this published article (and its “Supplementary Information” files).
