# Supplementary material for: Evolution of kaiA, a key circadian gene of cyanobacteria
Source: Sci Rep. 2021 May 11;11:9995. doi: 10.1038/s41598-021-89345-7 (PMC8113500; doi:10.1038/s41598-021-89345-7)
Supplement: Supplementary file 1 — Supplementary Information. [file 41598_2021_89345_MOESM1_ESM.pdf]

Evolution of *kaiA,* a key circadian gene of cyanobacteria

Volodymyr Dvornyk[1, *, #], Qiming Mei[2, 3, *]

[1] Department of Life Sciences, College of Science and General Studies, Alfaisal University, Riyadh 11533, Kingdom of Saudi Arabia

[2] Southern Marine Science and Engineering Guangdong Laboratory (Guangzhou), Guangzhou, People's Republic of China

[3] Key Laboratory of Vegetation Restoration and Management of Degraded Ecosystems, South China Botanical Garden, Chinese Academy of Sciences, Guangzhou, People's Republic of China

[*] The authors contributed equally to the paper

[#] Correspondence to vdvornyk@alfaisal.edu

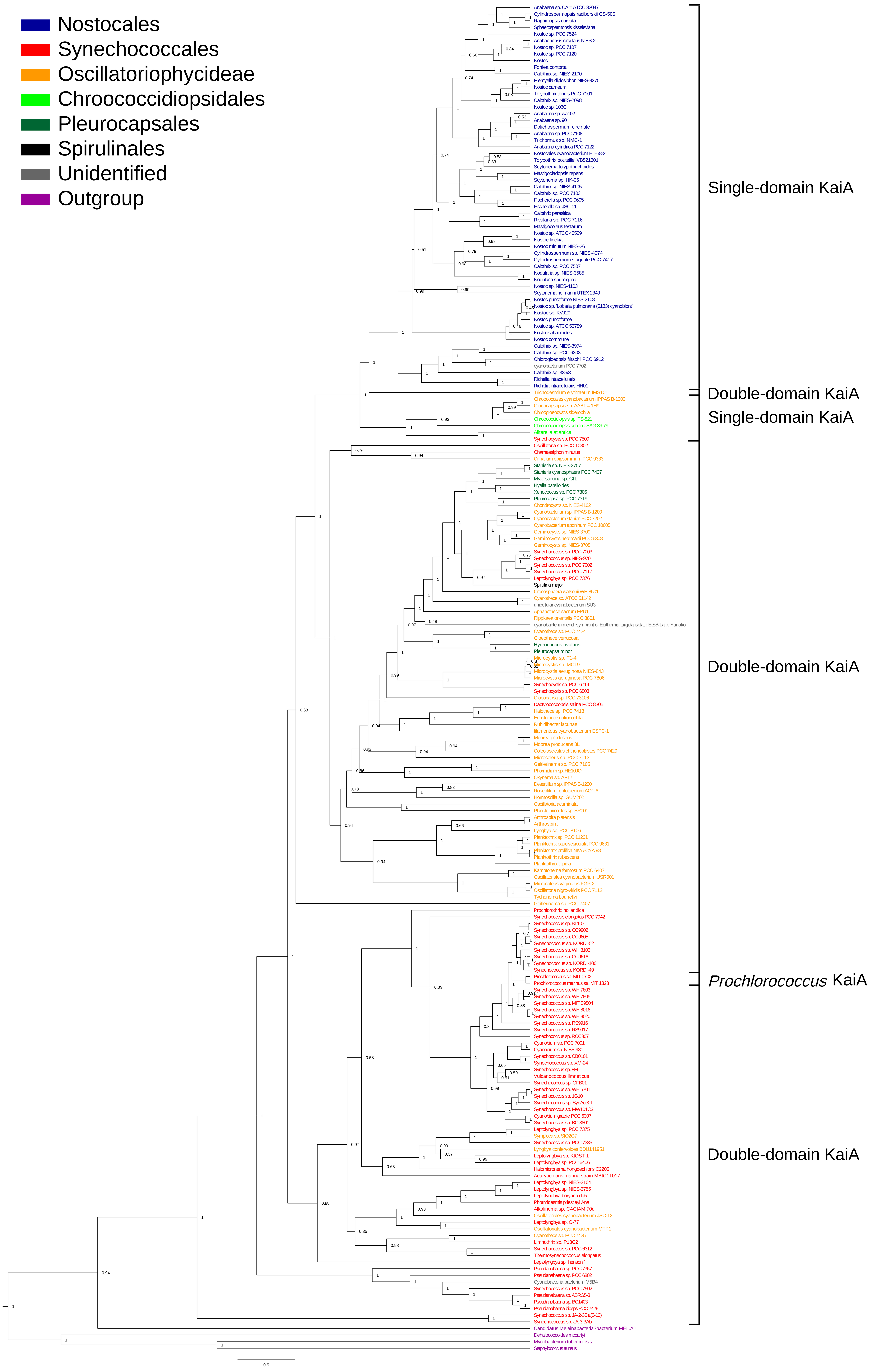

Figure S1. (a) Bayesian tree with uncorrelated log-normal clock of the 16S and 23S rRNA genes.

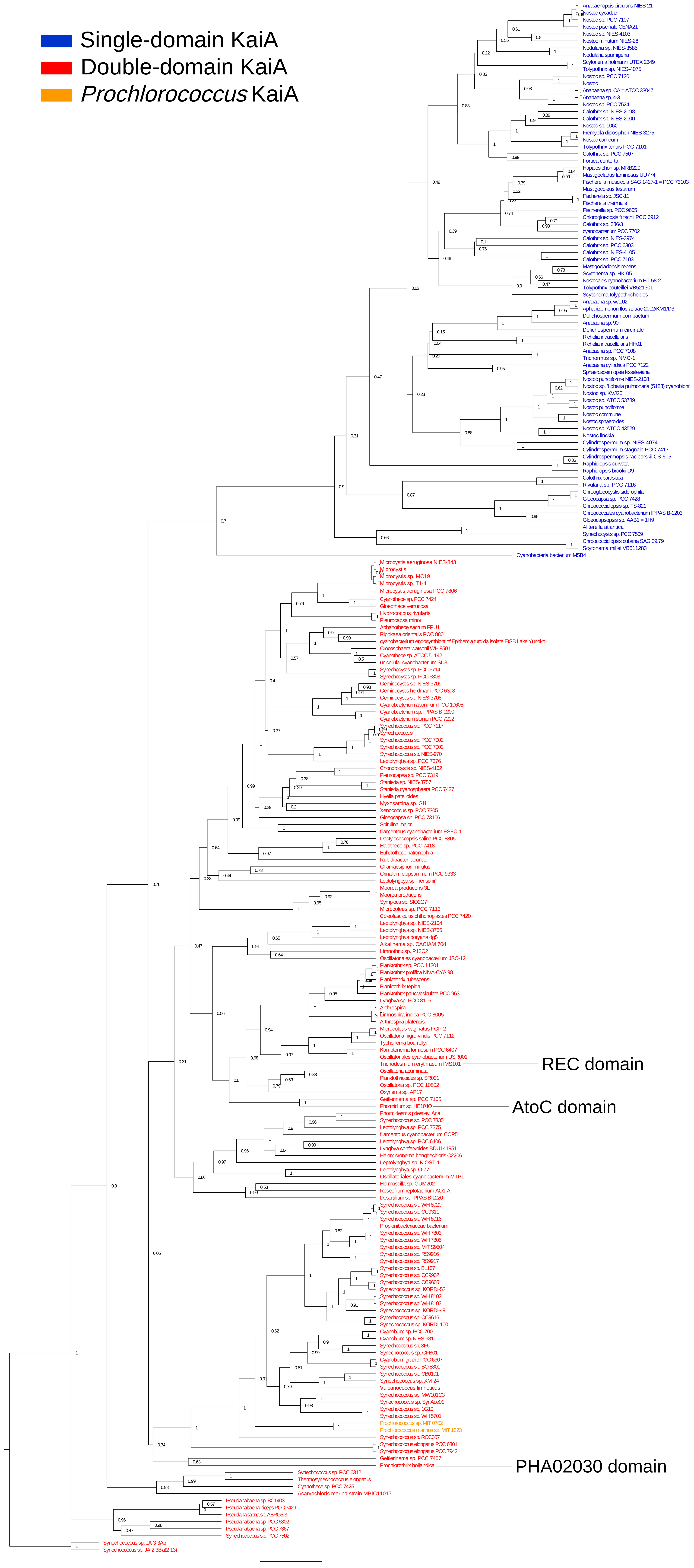

Figure S1. (b) Bayesian tree with uncorrelated log-normal clock of the KaiA proteins.

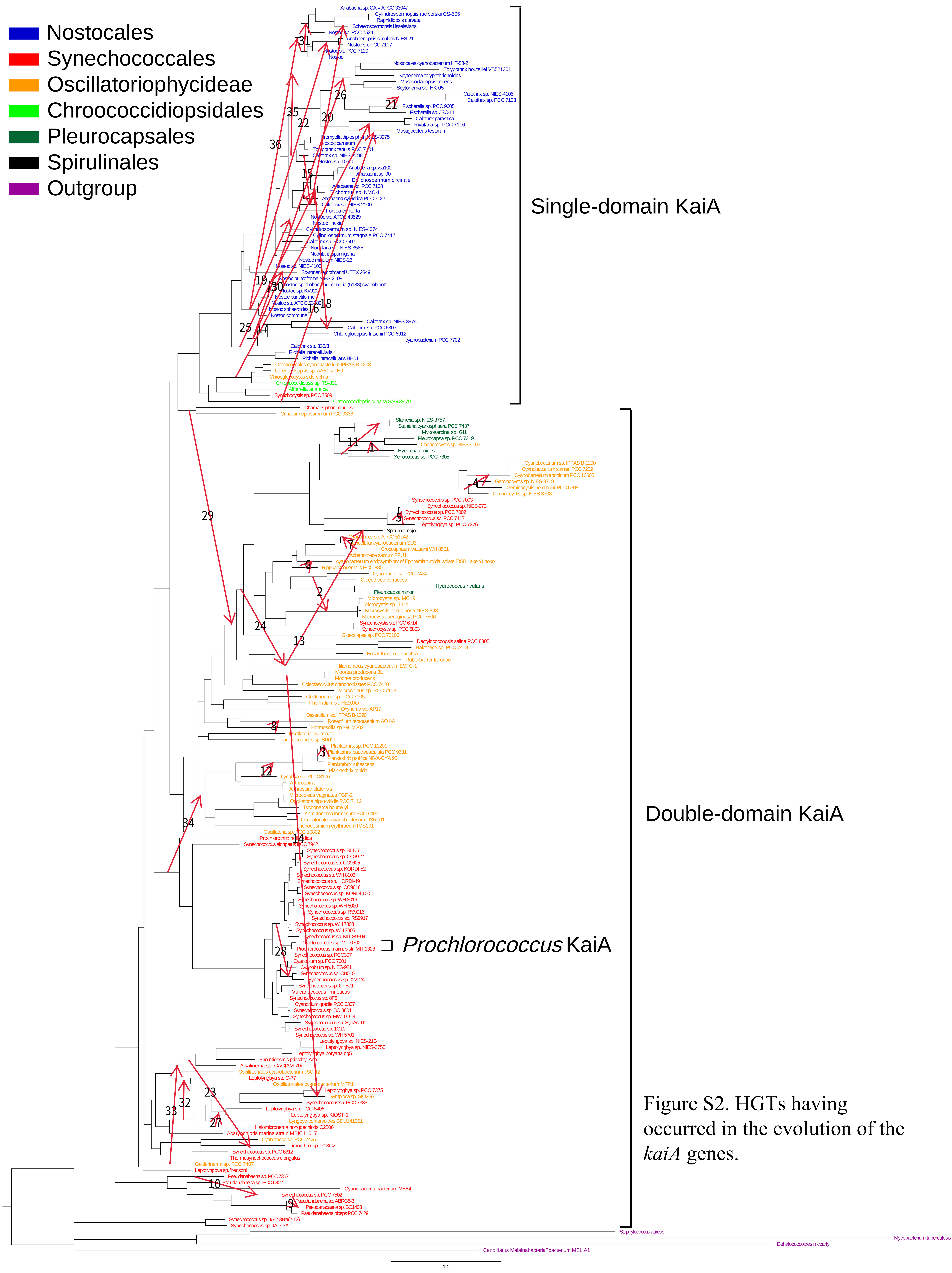

Figure S2. HGTs having occurred in the evolution of the *kaiA* genes.

Supplementary file 1. The results of the HGT analysis of the *kaiA* genes.

Species tree :

(((((((((((((((((((((Stani3757:0.00291100000000108,Stani7437:0.00783800000000000
12):0.06395099999999987,MyxosGI1:0.11468699999999998):0.0276929999999999
68,(Chond4102:0.05835199999999996,Pleur7319:0.05323699999999998):0.082036)
:0.009827999999999948,(Hyelpa:0.08323499999999995,Xenoc7305:0.07555299999
999998):0.025413999999999826):0.02667100000000011,(((Cyano1200:0.02055600
000000002,Cyano7202:0.015491999999999984):0.076654,Cyano10605:0.078376999
99999992):0.01504100000000082,((Gemin3709:0.02399600000000013,Gemin6308
:0.045130000000000114):0.01668000000000028,Gemin3708:0.0366150000000000
64):0.01736800000000005):0.254329):0.018439000000000094,(((((Synec7003:0.004
489999999999883,Synec970:0.032497):0.009419999999999984,Synec7002:0.00173
80000000000173):0.002433000000001295,Synec7117:0.001370000000000934):0.
02071299999999998,Lepto7376:0.05089300000000008):0.11526999999999998,Spir
uma:0.10639300000000018):0.02673900000000007):0.10151199999999982,(((((((C
yano51142:0.006550999999999735,uniceSU3:0.012839999999999963):0.00993299
999999997,Croco8501:0.07604499999999992):0.05456200000000022,AphanFPU1:
0.07254899999999997):0.015557999999999996,endosEpith:0.06407400000000019):0
.008454999999999988,Rippk8801:0.04609200000000002):0.03539800000000004,((C
yano7424:0.07580199999999992,Gloeoverr:0.053754999999999775):0.0668490000
0000016,(Hydrrivu:0.1404050000000001,Pleurmino:0.027511000000000063):0.1171
24):0.03214399999999995):0.013552000000000008,((((MicroMC19:0.00707099999
9999827,MicroT14:1.9999999998354667E-6):6.859999999999644E-
4,Micro843:0.003461999999999854):0.002432000000002118,Micro7806:4.920000
00000159E-
4):0.13094899999999976,(Synec6714:0.002852000000000767,Synec6803:0.00709
7000000000131):0.12413099999999977):0.03739700000000125):0.0208189999999
9992):0.019098999999999977,Gloeo73106:0.17144499999999985):0.013048000000
00017,((((Dacty8305:0.0629869999999999,Halot7418:0.06064499999999984):0.077
10100000000009,Euhalna:0.04874200000000006):0.11087099999999994,Rubila:0.2
3022600000000004):0.047655,filamESFC1:0.15581899999999993):0.023295000000
000066):0.02112999999999987,(((Moore3L:0.011036999999999964,Mooreprod:0.0
10691999999999924):0.13200100000000003,Coleo7420:0.08236599999999994):0.0
17715000000000147,Micro7113:0.1662920000000001):0.03294099999999989):0.02
484900000000012,((Geitl7105:0.06195799999999996,Phrom10JO:0.0674859999999
9993):0.08072000000000012,Oxyne17:0.20631):0.02274500000000015):0.0127009
99999999851,((Deser1220:0.11296300000000015,RoseoAO1A:0.152265000000000
1):0.022902999999999896,Horm202:0.14492799999999995):0.0424539999999999
):0.011436000000000002,(Oscilacum:0.13874399999999998,PlankSR001:0.1213839
9999999994):0.019245999999999874):0.009148000000000156,(((((Plank11201:0.01
0977000000000015,Plank9631:0.00538100000000008):0.005009999999999959,(Pla
nk98:2.000000000575113E-6,Plankrub:2.000000000575113E-
6):0.009643000000000068):0.028314999999999868,Planktepi:0.0405819999999998
96):0.12143700000000002,((Arthro:0.00507000000000013,ArthrPara:0.0037380000
00000241):0.07618399999999981,Lyngb8106:0.06448900000000002):0.009816999
999999965):0.05620200000000085,((((MicroFGP2:0.002177999999999791,Oscil7
112:0.003611999999999486):0.009393000000000207,Tychobou:0.036665000000000

0017):0.04538799999999984,(Kampt6407:0.023995999999999906,Oscil001:0.0172
10999999999865):0.06069700000000022):0.07071699999999992,Trich101:0.14069
100000000012):0.01850799999999997):0.02196700000000007):0.01697499999999999
962,Oscil10802:0.13808299999999996):0.025380999999999876,(((((((((((((((Nosto
582:0.05171099999999984,Tolyp1301:0.14355000000000007):0.00997000000000000
34,Scytotoly:0.07099299999999986):0.009341999999999961,(Mastirepe:0.05907200
000000001,ScytoHK05:0.052007000000000136):0.024938999999999822):0.035144
99999999998,((Calot4105:0.025354999999999794,Calot7103:0.03230299999999975
):0.1842330000000001,(Fisch9605:0.05275200000000013,FischJSC11:0.066150000
00000015):0.04537599999999986):0.024810999999999916):0.019411999999999985
,((Calotpara:0.012669000000000041,Rivul7116:0.00982100000000008):0.10145599
999999999,Mastitest:0.07887299999999997):0.052373):0.04603500000000005,(((((
Cylin505:0.004025000000000167,Raphicurv:0.006772):0.0627089999999999,Sphae
kiss:0.025359999999999827):0.03451700000000013,Anaba33047:0.0379110000000
0003):0.012561999999999962,Nosto7524:0.028993999999999964):0.013606999999
999925,(((AnabaN21:0.007156999999999858,Nosto7107:0.008375000000000021):0
.04759500000000054,Nosto7120:0.012524999999999897):0.009498000000000006,
Nostoc:0.03127400000000024):0.011333999999999955):0.010952000000000073):
0.009109999999999951,((((Fremy3275:0.011631999999999865,Nostocarn:0.011574
999999999891):0.011144000000000043,Tolyp7101:0.007215999999999889):0.0097
10000000000107,Calot2098:0.01699700000000015):0.004975999999999869,Nostoc
106:0.0325899999999999):0.011390000000000011):0.003278000000000114,(((((An
aba102:0.012044000000000166,Anaba90:0.026074000000000153):0.007383999999
999835,Doliccirc:0.025366999999999917):0.042153000000000011,(Anaba7108:0.018
945999999999907,TrichN1:0.014051999999999953):0.012634000000000034):0.012
855999999999979,Anaba7122:0.025781000000000054):0.020526000000000044,(Ca
lot2100:0.032374000000000125,Forticont:0.03991800000000012):0.0133950000000
00046):0.007476999999999956):0.014896999999999938,(Nosto43529:0.017195000
00000007,Nostolinc:0.020963999999999983):0.030696000000000057):0.007139000
0000000065,((Cylin4074:0.023009999999999975,Cylin7417:0.03551199999999999)
:0.012884999999999813,Calot7507:0.03673499999999996):0.010976000000000097
):0.001659999999999948,((Nodul3585:0.009519999999999973,Nodulspum:0.0089
32999999999858):0.04406599999999994,NostoN26:0.032467000000000024):0.003
1650000000000844):0.05744900000000008,(Nosto4103:0.03947199999999995,Scyt
o2349:0.08638699999999977):0.010778000000000176):0.004691999999999185,(((
(((Nosto2108:0.003149999999999986,NostoLoba:0.010273999999999894):0.005627
999999999966,NostoK20:0.011905999999999972):0.001792000000000158,Nostop
unc:0.003706999999999905):0.005006999999999984,Nosto53789:0.002191999999
999717):0.009118000000000182,Nostospha:0.006851000000001625):0.004043999
9999999365,Nostocomm:0.014151000000000025):0.03181499999999993):0.018140
000000000045,((((Calot3974:0.129513,Calot6303:0.09468199999999993):0.0439849
9999999994,(Chlor6912:0.04764899999999983,cyano7702:0.17896299999999998):
0.06525200000000009):0.018053000000000097,Calot3363:0.05250200000000005):
0.019223999999999908,(Riche:0.017242999999999897,Riche01:0.04135499999999
981):0.05169200000000007):0.02851300000000001):0.05856799999999995,(((((Chr
oo1203:0.014550999999999998,Gloeo1H9:0.013885000000000147):0.014185999999
99981,Chrooside:0.018681999999999865):0.006345000000000045,Chroo821:0.0368
6300000000009):0.06450200000000006,(Aliteatla:0.070832,Synec7509:0.04477699
9999999845):0.05337300000000115):0.01445300000000005,Chrooc3979:0.219737
00000000007):0.01670599999999888):0.03921100000000011,(Chamaminu:0.1900

710000000001,Crina9333:0.14634599999999987):0.03319000000000005):0.014681
999999999862):0.028625000000000123,((((((((((Synec107:5.710000000000992E-
4,Synec9902:5.770000000000497E-
4):0.012881999999999838,(Synec9605:0.003705999999999987,SynecK52:0.003279
9999999999496):0.0028569999999998874):0.006121000000000043,Synec8103:2.00
00000000575113E-
6):0.004210999999999965,SynecK49:0.005831999999999837):0.002763000000000
071,(Synec9616:0.0037730000000000263,SynecK100:0.005552000000000001):0.01
6820999999999975):0.006929000000000185,(((Synec8016:9.200000000000319E-
4,Synec8020:0.002539000000000069):0.008448000000000011,(Synec9916:0.01603
2000000000046,Synec9917:0.022670999999999886):0.011249000000000065):0.003
0879999999999797,((Synec7803:0.0031049999999999134,Synec7805:0.005081999
999999809):0.0021420000000000883,Synec9504:0.020415999999999999):0.0016540
000000000443):0.004717000000000082):0.008153999999999773,(Proch:0.0080690
00000000104,Proch1323:0.0012590000000001211):0.021356999999999985):0.01394
6000000000236,Synec307:0.032313000000000036):0.014489999999999892,((((Cya
no7001:0.003063000000000038,Cyano981:0.015710000000000113):0.01252699999
9999955,(Synec0101:0.006879999999999997,SynecXM24:0.020365000000000019):0
.021827999999999996):0.007020000000000026,((SynecGFB01:0.0229730000000001
32,Vulcalimn:0.011415000000000175):0.004368999999999845,Synec8F6:0.011320
999999999914):0.004231000000000096):0.007913999999999977,((((Synec1G10:0.0
01867000000000063,Synec5701:0.003992999999999913):0.006874000000000047,S
ynecAce01:0.027679999999999927):0.007779999999999898,Synec101C3:0.014207
00000000008):0.005397000000000096,(Cyano6307:0.004525000000000112,Synec8
801:0.002637000000000005):0.018351000000000006):0.010205999999999937):0.
014718000000000009):0.13145799999999985,Synec7942:0.085874):0.02361400000
0000024,Prochholl:0.138428999999999):0.046393000000000013):0.0184059999999
99922,(((((((Lepto2104:0.025868999999999992,Lepto3755:0.037793999999999988):0
.034588000000000006,Leptodg5:0.018863000000000074):0.119977,PhormAna:0.070
72499999999993):0.037339999999999993,Alkal70d:0.073291):0.0268540000000001
56,Oscil12:0.096638):0.014873999999999832,(LeptoO77:0.098573999999999994,Os
cilMTP1:0.142552):0.032451000000000001):0.031630000000000005,((((((Lepto7375:
0.030159999999999965,Symp2G7:0.039339000000000001):0.116891000000000008,S
ynec7335:0.113754000000000013):0.051012999999999975,(Lepto6406:0.065487999
99999999,LeptoK1:0.111429):0.025473000000000008):0.011983999999999995,Lyng
b1951:0.14517599999999997):0.020527999999999988,Halom2206:0.1035719999999
9978):0.044585000000000001,AcaryMBIC:0.096807000000000009):0.02564100000000
0025):0.007701999999999876,((Cyano7425:0.10349699999999995,Limno13C2:0.15
368099999999996):0.073425999999999999,(Synec6312:0.065947999999999999,Therm
elon:0.061879999999999935):0.057153999999999993):0.016897000000000162):0.01
3405000000000111):0.010240999999999945,(Geitl7407:0.087156000000000001,Lept
ohens:0.086799999999999999):0.008409000000000011):0.042551999999999992,((((((P
seud1403:0.017789000000000055,Pseud7429:0.009495000000000031):0.009034999
999999904,PseudG53:0.008099999999999996):0.101244000000000011,Synec7502:0.
083628000000000004):0.033374000000000015,cyanoM5B4:0.232452000000000001):0.
047243999999999984,Pseud6802:0.056391999999999776):0.036175000000000007,Ps
eud7367:0.10299999999999998):0.095311000000000015):0.0374785,((((Staph:0.6670
930000000002,Mycob:1.16699):0.097941999999999997,Dehal:1.052419):0.223487,
Melai:0.739460999999999999):0.229101999999999992,(Synec23Ba:0.03510900000000

006,Synec33Ab:0.0384880000000008):0.2398939999999983):0.037478500000000164);

Gene Tree :

((Synec23Ba:0.1322000000000001,Synec33Ab:0.10483299999999973):0.4171739999999999,(((Synec7502:0.5461069999999997,(Pseud6802:0.3553169999999999,Pseud7367:0.6095669999999997):0.12627299999999986):0.09175700000000031,(cyanoM5B4:2.8439540000000005,(Pseud7429:0.06435000000000013,(Pseud1403:0.06838200000000016,PseudG53:0.0997110000000001):0.012630999999999837):0.293307):0.1797439999999999):0.26782099999999964,((AcaryMBIC:0.4899479999999996,(Cyano7425:0.27981300000000076,(Synec6312:0.4700299999999995,Thermelon:0.4092720000000001):0.2559320000000005):0.11190399999999912):0.10226100000000038,((Coleo7420:0.24035100000000043,(Micro7113:0.21585699999999974,(Symp2G7:0.2443230000000005,(Moore3L:0.031393000000000004,Mooreprod:0.009848999999999997):0.3433830000000002):0.045220999999999734):0.0573990000000002):0.1803840000000001,((((LeptoO77:0.3559239999999999,OscilMTP1:0.3700289999999997):0.17413900000000027,(LeptoK1:0.8776920000000001,((Lepto6406:0.3848090000000002,Lyngb1951:0.501296):0.1349260000000001,(Halom2206:0.2629190000000001,(Lepto7375:0.36443599999999954,(PhormAna:0.26383899999999993,Synec7335:0.2427279999999996):0.18733500000000003):0.17924700000000016):0.04281700000000033):0.11429899999999993):0.17849499999999985):0.0652140000000001,(((Aliteatla:0.8213219999999999,Synec7509:0.7166809999999999):0.48656900000000025,(Chrooc3979:0.759792,Mastitest:0.984423):0.06510600000000011):0.06809799999999999,(((Calotpara:0.18047700000000022,Rivul7116:0.21242500000000009):0.8146499999999999,((Chroo1203:0.12584399999999984,Gloeo1H9:0.417354):0.1411340000000001,(Chroo821:0.10440000000000005,Chrooside:0.16507300000000003):0.277701):0.4924689999999998):0.20448600000000017,((Cylin505:0.015299000000000174,Raphicurv:0.11407999999999996):1.2933020000000002,((((Anaba7122:0.6117949999999999,Sphaekiss:0.29361099999999984):0.125432,(Doliccirc:0.34353299999999987,(Anaba102:0.21429799999999988,Anaba90:0.3257629999999998):0.2135349999999998):0.2346210000000002):0.03377000000000008,((Calot6303:0.8033869999999999,(Anaba7108:0.2524549999999999,TrichN1:0.10693300000000017):0.13903099999999968):0.029047000000000267,(Calot3974:1.0877909999999997,(Riche:0.42829300000000003,Riche01:0.41864800000000013):0.36844100000000024):0.24131100000000005):0.05886499999999945):0.0974070000000002,((Cylin4074:0.17703199999999963,Cylin7417:0.23665299999999956):0.13011700000000026,(((Nosto43529:0.2370770000000002,Nostolinc:0.12217199999999995):0.18564599999999976,((Nostocomm:0.07552700000000012,Nostospha:0.07948200000000005):0.08426400000000012,((Nosto53789:0.06140800000000013,Nostopunc:0.0102190000000002):0.07902399999999998,(NostoK20:0.09570200000000018,(Nosto2108:0.03602799999999995,NostoLoba:0.08365599999999995):0.06418500000000016):0.015013000000000165):0.049370000000000136):0.10226299999999977):0.13482299999999992,(((Calot7507:0.2529569999999999,Forticont:0.4052959999999999):0.04325599999999996,((Nostoc106:0.09737799999999996,(Calot2098:0.2082600000000001,Calot2100:0.35689899999999986):0.06697199999999981):0.05596800000000002,(Tolyp7101:0.04089000000000009,(Fremy3275:0.027388999999999886,Nostocarn:0.03858499999999987):0.05576099999999995):0.37275500000000017):0.12114499999999984):0.15390299999999968,((((Anaba33047:0.1270539999999997

8,Nosto7524:0.16546100000000008):0.09989200000000009,(Nosto7120:0.02224899
9999999963,Nostoc:0.25870099999999985):0.25558000000000014):0.23153800000
000002,((Nodul3585:0.18793900000000008,Nodulspum:0.04205099999999984):0.1
624150000000002,((AnabaN21:0.017443999999999793,Nosto7107:0.027041999999
99979):0.30296600000000007,(Nosto4103:0.36146900000000004,NostoN26:0.1241
9700000000011):1.9999999998354667E-
6):0.04256200000000021):0.02156799999999981):0.04863099999999987,(Scyto234
9:0.16295899999999985,(Scytotoly:0.36178500000000025,(((Mastirepe:0.14827499
999999993,ScytoHK05:0.07001800000000014):0.06230700000000011,(Nosto582:0.
08651300000000006,Tolyp1301:0.16400099999999984):0.015455999999999914):0.
05419600000000013,(FischJSC11:0.161451,((Calot3363:0.21580500000000002,(Chl
or6912:0.05130400000000016,cyano7702:0.461948):0.026746999999999854):0.08
144200000000001,(Fisch9605:0.02129000000000003,(Calot4105:0.13546600000000
01,Calot7103:0.37955799999999984):0.44719599999999999):0.08452899999999985)
:0.06096900000000005):0.14543600000000012):0.018191999999999986):0.103682
00000000005):0.05037599999999976):0.06064199999999964):0.153048000000000
07):0.040923000000000265):0.09063299999999996):0.14144500000000004):0.1267
8400000000023):0.10294899999999974):0.8150440000000003):0.037905999999999
55,(((Deser1220:0.30520999999999976,(Horm202:0.5851450000000002,RoseoAO1
A:0.4696060000000002):0.06444399999999995):0.12780199999999997,(Geitl7407:
0.5489410000000001,(Oscil12:0.35868000000000055,(Limno13C2:0.762937999999
9997,(Alkal70d:0.4812149999999997,(Leptodg5:0.223684,(Lepto2104:0.121676000
00000034,Lepto3755:0.1042080000000003):0.1885279999999998):0.152123999999
9997):0.07006200000000007):0.07114300000000018):0.07629100000000033):0.016
640999999999906):0.08330199999999977,((Leptohens:0.34864999999999924,((Cha
maminu:0.7253210000000005,Crina9333:0.37918300000000027):0.0977789999999
9917,((Rubila:1.1734100000000005,(Euhalna:0.24254700000000007,(Dacty8305:0.1
8603000000000014,Halot7418:0.1827380000000005):0.08260199999999962):0.428
70400000000064):0.15664899999999982,((filamESFC1:0.5213350000000001,Spiru
ma:0.38432099999999999):0.11648999999999976,((((Synec6714:0.03331000000000
017,Synec6803:0.016942000000000235):0.502173,((AphanFPU1:0.17056100000000
018,(endosEpith:0.43100199999999999,Rippk8801:0.10686399999999985):0.086972
00000000027):0.06999699999999986,(uniceSU3:0.11209399999999992,(Croco8501
:0.1342270000000001,Cyano51142:0.04802400000000029):0.005583000000000116
):0.17984699999999965):0.12146900000000027):0.023189000000000348,((Hydrriv
u:0.008944999999999759,Pleurmino:0.017742000000000147):0.2105120000000004
8,((Cyano7424:0.08378700000000006,Gloeoverr:0.13135600000000025):0.1189939
9999999982,(Micro7806:0.010930000000000106,(Micro843:0.01211699999999993
3,(MicroMC19:3.000000000419334E-
6,MicroT14:0.018438000000000176):0.006012999999999824):0.0043260000000002
74):0.32577199999999973):0.09444600000000047):0.023753000000000135):0.0327
9699999999952,((Lepto7376:0.29637199999999986,(Synec970:0.154473999999999
56,(Synec7003:0.04281800000000002,(Synec7002:0.05383000000000044,Synec71
17:1.9999999998354667E-
6):0.028713999999999906):0.07276399999999983):0.19018900000000016):0.30211
699999999997,(((Cyano1200:0.11298800000000009,Cyano7202:0.05872000000000
0105):0.2362719999999996,(Cyano10605:0.15698599999999985,(Gemin3708:0.091
364,(Gemin3709:0.053496999999999684,Gemin6308:0.08310899999999988):0.019
50900000000022):0.040455999999999825):0.09582599999999974):0.181414000000
0002,(Gloeo73106:0.38222500000000004,(MyxosGI1:0.5063360000000006,((Stani3

757:0.06168700000000005,Stani7437:0.04372599999999993):0.2219409999999997
2,(Xenoc7305:0.4918739999999997,(Hyelpa:0.5247669999999998,(Chond4102:0.33
799800000000024,Pleur7319:0.240383):0.26567799999999986):0.00857600000000
0139):0.06172699999999865):0.05271900000000626):0.1308239999999996):0.06
20339999999997):0.03203900000000015):0.00841400000000144):0.115784999999
9998):0.06343700000000041):0.08049699999999937):0.03113600000000053):0.05
3303000000000544,(((Trich101:0.5315129999999995,((Kampt6407:0.120953000000
00009,Oscil001:0.12470300000000023):0.16297099999999975,(Tychobou:0.090646
9999999997,(MicroFGP2:0.03357700000000019,Oscil7112:0.01275100000000623
):0.08736699999999953):0.22625299999999982):0.11280000000000001):0.0856640
000000004,((Arthro:0.004597999999999658,ArthrPara:0.023841):0.2936130000000
0057,(Lyngb8106:0.1620619999999997,(Plank9631:0.03711100000000034,(Plankte
pi:0.06237799999999982,(Plankrub:0.007152999999999743,(Plank11201:0.0205570
0000000016,Plank98:0.013417999999999708):0.05933399999999776):0.02054199
9999999838):0.023870000000000502):0.20015499999999964):0.0672380000000005
8):0.26936499999999963):0.046336000000000155,((Oxyne17:0.3652170000000003
5,(Oscil10802:0.31405499999999975,(Oscilacum:0.28553600000000046,PlankSR00
1:0.30615400000000026):0.04036799999999907):0.05897200000000069):0.062663
99999999983,((Geitl7105:0.2553149999999995,Phrom10JO:0.4338619999999995):
0.15793300000000077,(Prochholl:0.72342,(Synec7942:0.5277289999999999,(Synec
307:0.37742900000000024,((Cyano6307:1.9999999998354667E-
6,Synec8801:0.03708899999999993):0.26613,(((Cyano7001:0.041125999999999774
,Cyano981:0.07250200000000007):0.3115349999999997,(Synec8F6:0.10319799999
99999,SynecGFB01:0.22531200000000018):0.10493899999999945):0.04502500000
000076,(((Synec101C3:0.2519629999999994,SynecAce01:0.34445000000000014):
0.04837399999999992,(Synec1G10:0.04892699999999994,Synec5701:0.056096000
000000146):0.30680700000000005):0.05615599999999965,((Vulcalimn:0.20490799
999999965,(Synec0101:0.12270200000000031,SynecXM24:0.1947689999999997):
0.12335199999999968):0.11414400000000047,(((Synec9616:0.04909300000000005,
SynecK100:0.019974999999999632):0.05167700000000195,((Synec8103:0.155136
00000000016,SynecK49:0.1255980000000001):0.02835600000000048,((Synec107:
0.013866000000000156,Synec9902:0.01455200000000012):0.11981699999999984,(
Synec9605:0.019883999999999979,SynecK52:0.03740000000000032):0.1004290000
000001):0.06801199999999996):0.0644610000000001):0.11901300000000026,((Syn
ec9916:0.14998999999999985,Synec9917:0.07970100000000002):0.1135489999999
999,((Propibact:0.05105499999999985,(Synec8016:0.011261000000000188,Synec80
20:0.02008900000000024):0.02477399999999985):0.09614300000000053,((Synec
7803:0.06898800000000005,Synec7805:0.021587999999999496):0.0785810000000
0023,(Synec9504:2.000000000279556E-
6,(Proch:0.31686999999999976,Proch1323:0.8813459999999997):2.692582):0.0888
9900000000006):0.08973900000000024):0.02581599999999984):0.04819800000000
0185):0.23095700000000008):0.028340999999999283):0.06084000000000067):0.04
241199999999967):0.19641200000000048):0.7186269999999997):0.2480310000000
001):0.03929699999999947):0.0924820000000004):0.009967999999999755):0.0580
4700000000018):0.01857400000000009):0.016307000000000293):0.0434529999999
9952):0.06604200000000038):0.2655560000000001):0.417174);

Criteria values before the computation :

Robinson and Foulds distance (RF) = 218

Least-squares coefficient(LS) = 285.010

Bipartition dissimilarity = 443.0

Bootstrap values were computed with 1 gene trees

HGT results:

1 Regular (bootstrap value = 100.0% inverse = 100.0%)

From subtree (Hyelpa) to subtree (Chond4102, Pleur7319)

RF = 214 , LS = 281.052 , BD = 439.5

rRF = 216 , rLS = 281.096 , rBD = 440.5

2 Regular (bootstrap value = 100.0% inverse = 100.0%)

From subtree (Cyano7424, Gloeoverr) to subtree (Micro7806, Micro843, MicroMC19, MicroT14)

RF = 214 , LS = 280.745 , BD = 437.0

rRF = 216 , rLS = 280.764 , rBD = 439.0

3 Regular (bootstrap value = 100.0% inverse = 100.0%)

From subtree (Plank98) to subtree (Plank11201)

RF = 214 , LS = 281.091 , BD = 440.0

rRF = 216 , rLS = 281.102 , rBD = 441.0

4 Regular (bootstrap value = 100.0% inverse = 100.0%)

From subtree (Gemin3708, Gemin3709, Gemin6308) to subtree (Cyano10605)

RF = 216 , LS = 281.044 , BD = 442.0

rRF = 216 , rLS = 281.096 , rBD = 442.0

5 Regular (bootstrap value = 100.0% inverse = 100.0%)

From subtree (Synec7117) to subtree (Synec7002)

RF = 216 , LS = 281.105 , BD = 441.5

rRF = 216 , rLS = 281.116 , rBD = 441.5

6 Regular (bootstrap value = 100.0% inverse = 100.0%)

From subtree (Rippk8801) to subtree (endosEpith)

RF = 216 , LS = 281.046 , BD = 439.5

rRF = 216 , rLS = 281.055 , rBD = 439.5

7 Regular (bootstrap value = 100.0% inverse = 100.0%)

From subtree (Croco8501) to subtree (Cyano51142)

RF = 216 , LS = 281.122 , BD = 442.0

rRF = 216 , rLS = 281.122 , rBD = 442.0

8 Regular (bootstrap value = 100.0% inverse = 100.0%)

From subtree (Horm202) to subtree (RoseoAO1A)

RF = 216 , LS = 281.170 , BD = 442.0

rRF = 216 , rLS = 281.113 , rBD = 442.0

9 Regular (bootstrap value = 100.0% inverse = 100.0%)

From subtree (PseudG53) to subtree (Pseud1403)

RF = 216 , LS = 281.226 , BD = 442.0

rRF = 216 , rLS = 281.122 , rBD = 442.0

10 Regular (bootstrap value = 100.0% inverse = 100.0%)

From subtree (Pseud6802, Pseud7367) to subtree (Synec7502)

RF = 172 , LS = 278.762 , BD = 399.5

rRF = 174 , rLS = 279.169 , rBD = 400.5

11 Regular (bootstrap value = 100.0% inverse = 100.0%)

From subtree (Chond4102, Hyelpa, Pleur7319, Xenoc7305) to subtree (Stani3757, Stani7437)

RF = 174 , LS = 279.018 , BD = 400.5

rRF = 174 , rLS = 279.043 , rBD = 400.5

12 Regular (bootstrap value = 100.0% inverse = 100.0%)

From subtree (Lyngb8106) to subtree (Plank11201, Plank9631, Plank98, Plankrub, Planktepi)

RF = 160 , LS = 278.588 , BD = 392.0

rRF = 160 , rLS = 278.585 , rBD = 392.0

13 Regular (bootstrap value = 100.0% inverse = 100.0%)

From subtree (filamESFC1) to subtree (Spiruma)

RF = 148 , LS = 276.880 , BD = 383.0

rRF = 150 , rLS = 278.965 , rBD = 385.0

14 Regular (bootstrap value = 100.0% inverse = 100.0%)

From subtree (Moore3L, Mooreprod) to subtree (Symp2G7)

RF = 150 , LS = 274.225 , BD = 378.5

rRF = 152 , rLS = 280.402 , rBD = 391.5

15 Regular (bootstrap value = 100.0% inverse = 100.0%)

From subtree (Calot2098) to subtree (Calot2100)

RF = 150 , LS = 274.127 , BD = 381.0

rRF = 152 , rLS = 279.801 , rBD = 386.5

16 Regular (bootstrap value = 100.0% inverse = 100.0%)

From subtree (Chrooc3979) to subtree (Mastitest)

RF = 152 , LS = 243.493 , BD = 377.0

rRF = 152 , rLS = 310.073 , rBD = 391.5

17 Regular (bootstrap value = 100.0% inverse = 100.0%)

From subtree (Chroo1203, Chroo821, Chrooside, Gloeo1H9) to subtree (Calotpara, Rivul7116)

RF = 152 , LS = 191.594 , BD = 362.0

rRF = 154 , rLS = 344.629 , rBD = 395.5

18 Regular (bootstrap value = 100.0% inverse = 100.0%)

From subtree (Anaba7108, TrichN1) to subtree (Calot6303)

RF = 152 , LS = 275.052 , BD = 380.0

rRF = 154 , rLS = 277.622 , rBD = 381.5

19 Regular (bootstrap value = 100.0% inverse = 100.0%)

From subtree (Nosto2108, Nosto53789, NostoK20, NostoLoba, Nostocomm, Nostopunc, Nostospha) to subtree (Nosto43529, Nostolinc)

RF = 152 , LS = 276.150 , BD = 374.0

rRF = 152 , rLS = 276.200 , rBD = 367.5

20 Regular (bootstrap value = 100.0% inverse = 100.0%)

From subtree (Anaba7122) to subtree (Sphaekiss)

RF = 152 , LS = 277.588 , BD = 379.0

rRF = 152 , rLS = 278.197 , rBD = 387.5

21 Regular (bootstrap value = 100.0% inverse = 100.0%)

From subtree (Fisch9605) to subtree (Calot4105, Calot7103)

RF = 152 , LS = 278.158 , BD = 384.0

rRF = 152 , rLS = 278.112 , rBD = 384.0

22 Regular (bootstrap value = 100.0% inverse = 100.0%)

From subtree (Nosto4103, NostoN26) to subtree (AnabaN21, Nosto7107)

RF = 90 , LS = 163.884 , BD = 275.0

rRF = 92 , rLS = 157.978 , rBD = 267.5

23 Regular (bootstrap value = 100.0% inverse = 100.0%)

From subtree (Alkal70d, Lepto2104, Lepto3755, Leptodg5) to subtree (Limno13C2)

RF = 90 , LS = 161.769 , BD = 273.5

rRF = 92 , rLS = 162.341 , rBD = 273.5

24 Regular (bootstrap value = 100.0% inverse = 100.0%)

From subtree (AphanFPU1, Chond4102, Croco8501, Cyano10605, Cyano1200, Cyano51142, Cyano7202, Cyano7424, Gemin3708, Gemin3709, Gemin6308, Gloeo73106, Gloeoverr, Hydrrivu, Hyelpa, Lepto7376, Micro7806, Micro843, MicroMC19, MicroT14, MyxosGI1, Pleur7319, Pleurmino, Rippk8801, Stani3757, Stani7437, Synec6714, Synec6803, Synec7002, Synec7003, Synec7117, Synec970, Xenoc7305, endosEpith, uniceSU3) to subtree (Spiruma, filamESFC1)

RF = 92 , LS = 162.247 , BD = 274.5

rRF = 92 , rLS = 162.915 , rBD = 274.5

25 Regular (bootstrap value = 100.0% inverse = 100.0%)

From subtree (Calot3974, Riche, Riche01) to subtree (Anaba7108, Calot6303, TrichN1)

RF = 68 , LS = 123.428 , BD = 225.0

rRF = 68 , rLS = 136.164 , rBD = 230.5

26 Regular (bootstrap value = 100.0% inverse = 100.0%)

From subtree (Calot3363, Calot4105, Calot7103, Chlor6912, Fisch9605, FischJSC11, cyano7702) to subtree (Mastirepe, Nosto582, ScytoHK05, Tolyp1301)

RF = 70 , LS = 132.013 , BD = 238.5

rRF = 70 , rLS = 132.433 , rBD = 238.5

27 Regular (bootstrap value = 100.0% inverse = 100.0%)

From subtree (Halom2206, Lepto7375, PhormAna, Synec7335) to subtree (Lepto6406, Lyngb1951)

RF = 70 , LS = 131.858 , BD = 238.5

rRF = 70 , rLS = 131.867 , rBD = 238.5

28 Regular (bootstrap value = 100.0% inverse = 100.0%)

From subtree (Proch, Proch1323, Synec107, Synec7803, Synec7805, Synec8016, Synec8020, Synec8103, Synec9504, Synec9605, Synec9616, Synec9902, Synec9916, Synec9917, SynecK100, SynecK49, SynecK52) to subtree (Synec0101, SynecXM24, Vulcalimn)

RF = 58 , LS = 94.356 , BD = 192.0

rRF = 58 , rLS = 89.394 , rBD = 191.5

29 Regular (bootstrap value = 100.0% inverse = 100.0%)

From subtree (Chamaminu, Crina9333) to subtree (AphanFPU1, Chond4102, Croco8501, Cyano10605, Cyano1200, Cyano51142, Cyano7202, Cyano7424, Dacty8305, Euhalna, Gemin3708, Gemin3709, Gemin6308, Gloeo73106, Gloeoverr, Halot7418, Hydrrivu, Hyelpa, Lepto7376, Micro7806, Micro843, MicroMC19, MicroT14, MyxosGI1, Pleur7319, Pleurmino, Rippk8801, Rubila, Spiruma, Stani3757, Stani7437, Synec6714, Synec6803, Synec7002, Synec7003, Synec7117, Synec970, Xenoc7305, endosEpith, filamESFC1, uniceSU3)

RF = 60 , LS = 94.974 , BD = 199.5

rRF = 60 , rLS = 93.530 , rBD = 197.0

30 Regular (bootstrap value = 100.0% inverse = 100.0%)

From subtree (Calot3363, Calot4105, Calot7103, Chlor6912, Fisch9605, FischJSC11, Mastirepe, Nosto582, ScytoHK05, Scytotoly, Tolyp1301, cyano7702) to subtree (Scyto2349)

RF = 60 , LS = 95.008 , BD = 195.0

rRF = 60 , rLS = 95.423 , rBD = 195.0

31 Regular (bootstrap value = 100.0% inverse = 100.0%)

From subtree (Nosto7120, Nostoc) to subtree (Anaba33047, Nosto7524)

RF = 60 , LS = 93.929 , BD = 198.0

rRF = 60 , rLS = 93.926 , rBD = 198.0

32 Regular (bootstrap value = 100.0% inverse = 100.0%)

From subtree (Halom2206, Lepto6406, Lepto7375, LeptoK1, Lyngb1951, PhormAna, Synec7335) to subtree (LeptoO77, OscilMTP1)

RF = 60 , LS = 95.755 , BD = 198.0

rRF = 60 , rLS = 95.964 , rBD = 200.5

33 Regular (bootstrap value = 100.0% inverse = 100.0%)

From subtree (Geitl7407) to subtree (Alkal70d, Lepto2104, Lepto3755, Leptodg5, Limno13C2, Oscil12)

RF = 62 , LS = 96.295 , BD = 198.0

rRF = 62 , rLS = 96.053 , rBD = 199.5

34 Regular (bootstrap value = 100.0% inverse = 100.0%)

From subtree (Cyano6307, Cyano7001, Cyano981, Geitl7105, Oscil10802, Oscilacum, Oxyne17, Phrom10JO, PlankSR001, Proch, Proch1323, Prochholl, Synec0101, Synec101C3, Synec107, Synec1G10, Synec307, Synec5701, Synec7803, Synec7805, Synec7942, Synec8016, Synec8020, Synec8103, Synec8801, Synec8F6, Synec9504, Synec9605, Synec9616, Synec9902, Synec9916, Synec9917, SynecAce01, SynecGFB01, SynecK100, SynecK49, SynecK52, SynecXM24, Vulcalimn) to subtree (ArthrPara, Arthro, Kampt6407, Lyngb8106, MicroFGP2, Oscil001, Oscil7112, Plank11201, Plank9631, Plank98, Plankrub, Planktepi, Trich101, Tychobou)

RF = 22 , LS = 54.614 , BD = 95.5

rRF = 22 , rLS = 54.704 , rBD = 95.5

35 Regular (bootstrap value = 100.0% inverse = 100.0%)

From subtree (Calot2098, Calot2100, Calot7507, Forticont, Fremy3275, Nostoc106, Nostocarn, Tolyp7101) to subtree (Anaba33047, AnabaN21, Calot3363, Calot4105, Calot7103, Chlor6912, Fisch9605, FischJSC11, Mastirepe, Nodul3585, Nodulspum, Nosto4103, Nosto582, Nosto7107, Nosto7120, Nosto7524, NostoN26, Nostoc, Scyto2349, ScytoHK05, Scytotoly, Tolyp1301, cyano7702)

RF = 18 , LS = 30.584 , BD = 91.5

rRF = 18 , rLS = 30.374 , rBD = 91.5

36 Regular (bootstrap value = 100.0% inverse = 100.0%)

From subtree (Nosto2108, Nosto43529, Nosto53789, NostoK20, NostoLoba, Nostocomm, Nostolinc, Nostopunc, Nostospha) to subtree (Anaba33047, AnabaN21,

Calot2098, Calot2100, Calot3363, Calot4105, Calot7103, Calot7507, Chlor6912, Fisch9605, FischJSC11, Forticont, Fremy3275, Mastirepe, Nodul3585, Nodulspum, Nosto4103, Nosto582, Nosto7107, Nosto7120, Nosto7524, NostoN26, Nostoc, Nostoc106, Nostocarn, Scyto2349, ScytoHK05, Scytotoly, Tolyp1301, Tolyp7101, cyano7702)

RF = 14 , LS = 17.092 , BD = 33.0

rRF = 14 , rLS = 16.630 , rBD = 33.0
